# Aberrant basal ganglia metabolism in fragile X syndrome: a magnetic resonance spectroscopy study

**DOI:** 10.1186/1866-1955-5-20

**Published:** 2013-08-28

**Authors:** Jennifer Lynn Bruno, Elizabeth Walter Shelly, Eve-Marie Quintin, Maryam Rostami, Sweta Patnaik, Daniel Spielman, Dirk Mayer, Meng Gu, Amy A Lightbody, Allan L Reiss

**Affiliations:** 1 Center for Interdisciplinary Brain Sciences Research, Stanford University, 401 Quarry Road, Stanford, CA 94305-5795, USA; 2 Department of Radiology, Stanford University, 300 Pasteur Drive, Stanford, CA 94305-5105, USA; 3 Neuroscience Program, SRI International, 333 Ravenswood Avenue, Menlo Park, CA 94025-3493, USA; 4 Department of Pediatrics, Stanford University, 300 Pasteur Drive, Stanford, CA 94305-5208, USA

**Keywords:** Fragile X syndrome, Magnetic resonance spectroscopy, Choline, Glutamate, Caudate nucleus

## Abstract

**Background:**

The profile of cognitive and behavioral variation observed in individuals with fragile X syndrome (FXS), the most common known cause of inherited intellectual impairment, suggests aberrant functioning of specific brain systems. Research investigating animal models of FXS, characterized by limited or lack of fragile X mental retardation protein, (FMRP), has linked brain dysfunction to deficits in the cholinergic and glutamatergic systems. Thus, we sought to examine *in vivo* levels of neurometabolites related to cholinergic and glutamatergic functioning in males and females with FXS.

**Methods:**

The study participants included 18 adolescents and young adults with FXS, and a comparison group of 18 individuals without FXS matched for age, sex and general intellectual functioning. Proton magnetic resonance spectroscopy (MRS) was used to assess neurometabolite levels in the caudate nucleus, a region known to be greatly enlarged and involved in abnormal brain circuitry in individuals with FXS. A general linear model framework was used to compare group differences in metabolite concentration.

**Results:**

We observed a decrease in choline (*P* = 0.027) and in glutamate + glutamine (*P* = 0.032) in the caudate nucleus of individuals with FXS, relative to individuals in the comparison group.

**Conclusions:**

This study provides evidence of metabolite differences in the caudate nucleus, a brain region of potential importance to our understanding of the neural deficits underlying FXS. These metabolic differences may be related to aberrant receptor signaling seen in animal models. Furthermore, identification of the specific neurometabolites involved in FXS dysfunction could provide critical biomarkers for the design and efficacy tracking of disease-specific pharmacological treatments.

## Background

Fragile X syndrome (FXS), the most common cause of inherited intellectual disability, affects approximately 1 in 5,000 males and 1 in 10,000 females [1]. FXS results from a trinucleotide CGG repeat expansion on the long arm of the X chromosome (locus Xq27.3) [2], hypermethylation of the fragile X mental retardation 1 (*FMR1*) gene promoter region and reduced production of the fragile X mental retardation protein (FMRP). Reduced FMRP expression has been linked to increased density of immature dendritic spines and abnormal dendritic morphology in humans with FXS [3] and mouse models of FXS (*Fmr1*-knockout (KO) mouse) [4]. FMRP is implicated in a variety of neurobiological functions, including the mammalian target of rapamycin (mTOR) [5] and the extracellular signal-regulated kinase 1/2 (ERK1/2) pathways [6].

Reduced FMRP results in a constellation of behavioral and cognitive impairments, including specific weaknesses in social cognition, communication and executive function [7-9], in addition to neurological abnormalities. One of the most replicated neuroanatomical findings is greatly and bilaterally enlarged caudate nucleus in FXS [10-12]. The caudate, via connections with the frontal lobe, is involved in impulse control and attention [13], key executive functions known to be deficient in individuals with FXS [9]. Accordingly, recent functional magnetic resonance imaging (fMRI) research in individuals with FXS has found evidence for alterations in the frontostriatal circuitry underlying executive function skills, including working memory and attention/inhibition [14].

Other neuroimaging research implicates specific neurotransmitter systems involving choline, glutamine and gamma-aminobutyric acid (GABA) [15,16]. Research examining metabolic systems in FXS has burgeoned following the finding that silencing FMRP in the *Fmr1*-KO mouse results in amplified signaling through specific G protein coupled receptors (GPCRs) – group I metabotropic glutamate receptors (mGluR1 and mGluR5) [17] and muscarinic acetylcholine receptors (mAChRs) [18]. Potential therapeutic interventions have been suggested based on genetic and pharmacological manipulations, which regulate GPCR signaling in *Fmr1*-KO mice, and subsequently result in reduction of some maladaptive behaviors associated with FXS [18,19]. Identification of affected brain systems in humans with FXS can provide links between the direct biological consequences of FMRP silencing and the neurobiological/behavioral/cognitive phenotypes of FXS, as well as provide endpoints for monitoring pharmacological intervention.

To date, examination of specific brain systems in humans with FXS is very limited. One *in vivo* investigation of neurometabolite levels in males with FXS reported reduced choline/creatine ratios in bilateral dorsolateral prefrontal cortex [15], an integral part of the corticostriatal executive functioning network in which aberrant functioning has been demonstrated in humans with FXS [14].

The present study sought to examine neurometabolite levels in a broader sample of individuals with FXS, including both females and males, to address the hypothesis that similar neurometabolic profiles are present in both sexes. Females, like males with fragile X syndrome, have reduced FMRP, and disadvantageous cognitive and behavioral symptoms, albeit to a lesser degree than their male counterparts [20]. Furthermore, structural brain abnormalities, including enlarged caudate nucleus, are present in both males and females with FXS, although some reports indicate less severe abnormalities for females [10,21-23]. An innovative component of the current study is that individuals with FXS were compared to individuals without FXS matched for age, sex and general intellectual functioning. Thus significant differences observed in neurometabolite profiles would be primarily linked to FXS and not cognitive functioning in general.

We examined the caudate nucleus because previous evidence has indicated this region’s importance to our understanding of the neurobiological basis of FXS [10-12,14,24]. Metabolic concentrations for the major proton metabolites were estimated with *in vivo* single-voxel proton magnetic resonance spectroscopy (MRS) and included *N*-acetylaspartate (NAA), creatine, choline, *myo*-inositol, glutamate, and glutamine + glutamate (Glx). We hypothesized that individuals with FXS, including females, would display lower levels of choline and glutamate-related metabolites relative to the comparison group.

## Methods

The participants included 27 adolescents and young adults with FXS (confirmed via evidence of full *FMR1* mutation on DNA testing utilizing standard Southern blot techniques; mean age = 20.79 years, SD = 3.38, 18 females), and a comparison group of 24 individuals without FXS (confirmed via genetic screening [25]; mean age = 19.64 years, SD = 2.82, 13 females). Participants in the comparison group were diagnosed with idiopathic developmental delay, intellectual disability or learning disability, and were matched to the FXS group for age, sex and general intellectual level (*P*s >0.10). Potential participants in the comparison group were excluded for any other known genetic condition, premature birth, low birth weight, or a history of severe psychiatric, neurological or medical disorder. Participants were free from magnetic resonance imaging (MRI) contraindications. Participants were recruited across the USA and Canada through advertisements, referrals, word of mouth and from our database. Participants and/or their parents gave written informed consent and assent to participate in the study. All protocols were approved by the Institutional Review Board at Stanford University, CA, USA.

Participant medications were grouped into three classes: 1) stimulants; 2) selective serotonin reuptake inhibitors (SSRIs); and 3) atypical antipsychotics, anticonvulsants and other drugs affecting neurological functioning. The association of medication class with metabolite concentration was assessed via multiple regression, separately in each participant group. One participant in the FXS group was taking the acetylcholinesterase inhibitor donepezil. Given this drug’s intended effect on the cholinergic system we considered this participant a potential outlier for all subsequent analyses.

General intellectual function was assessed via the Wechsler Abbreviated Scale of Intelligence (age 17 years or older) [26] or the Wechsler Intelligence Scale for Children (age younger than 17 years) [27]. Executive functioning was assessed via the Contingency Naming Test (CNT), a measure of processing speed and inhibition [28]. Parent ratings included the Aberrant Behavior Checklist [29] which measures problem behaviors and the Achenbach Adult or Child Behavior Checklist attention subscale which measures attention problems [30,31].

**Figure 1:**
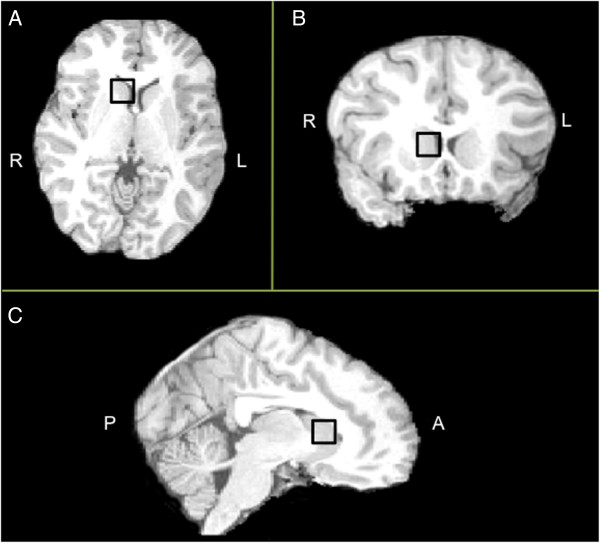
**Example voxel placement.** MRS voxel (black square) displayed on high resolution T1-weighted image for one example participant (from the comparison group). **(A)** Axial, **(B)** coronal and **(C)** sagittal. We placed 1.5 cm isotropic voxels over the head of the right caudate nucleus. Voxels were placed to maximize caudate head tissue. Note: original voxel was positioned on T2-weighted image, but high resolution T1-weighted image is shown here for enhanced resolution in sagittal and coronal planes. A, anterior; I, inferior; L, left; MRS, magnetic resonance spectroscopy; P, posterior; R, right; S, superior.

Participants were scanned on a 3 Tesla MRI Scanner (GE Signa, Milwaukee, WI, USA) at the Lucas Center for Neuroimaging, Stanford University, using one of two custom single-channel quadrature head coils (one head coil was decommissioned midway through the study). A T2-weighted gradient echo spiral pulse sequence (TE1 = 17 ms, TE2 = 85.0 ms, TR = 4000 ms, FOV = 24 cm, slice thickness = 5 mm, gap = 0 mm, slices = 19, frequency x phase = 256 × 192) was used to produce an image on which a 1.5 cm isotropic voxel was prescribed, encompassing as much of the right caudate head as possible (Figure 1). Given the voxel’s cubic shape and its size (larger than the caudate head) the voxel included portions of the adjacent lateral ventricle, the surrounding periventricular and frontal white matter, and the anterior aspect of the putamen. Due to scanning duration limitations we were only able to examine one region of interest and we chose the right hemisphere given its strong implication in FXS-deficient executive functioning networks [14].

**Figure 2:**
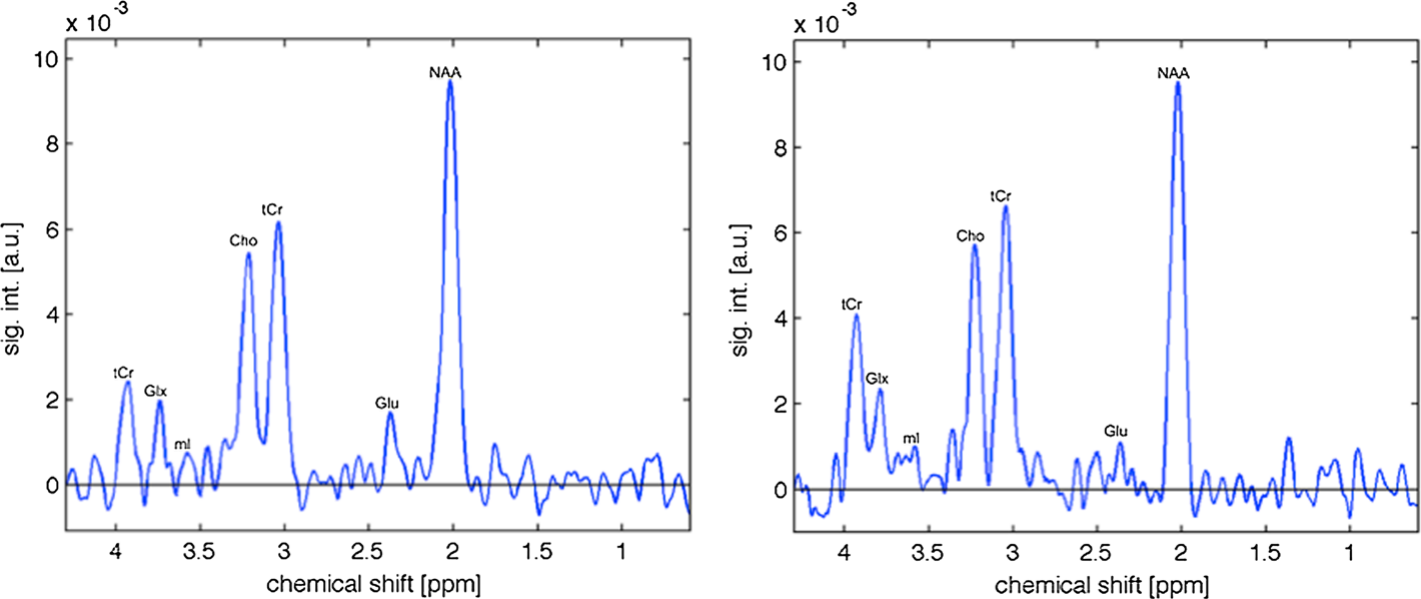
**Example spectra.** Example spectra for one individual from the comparison group (left) and one individual from the FXS group (right). Signal is in arbitrary units. Cho, choline; Glu, glutamate; Glx, glutamine + glutamate; mI, *myo*-inositol; NAA, *N*-acetylaspartate; tCr, creatine.

Single-voxel MRS of this region was acquired via constant-time point-resolved spectroscopy (CT-PRESS; average TE = 139 ms, n_1_ = 129, ∆t_½_ = 0.8 ms) [32]. The resulting spectra were analyzed with MATLAB (Natick, MA, USA) as described previously [33]. Metabolite signals, determined by peak integration (with an interval of ±6 Hz), included the major proton metabolites, NAA (2.01 ppm), creatine (3.03 and 3.93 ppm), choline (3.24 ppm), *myo*-inositol (3.58 ppm), glutamate (2.36 ppm) and Glx (3.78 ppm) (Figure 2). An acquisition was acquired without water suppression to measure tissue water content (including cerebrospinal fluid (CSF)), which was then used to normalize concentrations of each metabolite thus accounting for tissue fraction in the voxel [33,34].

Spectra were included only if the signal-to-noise ratio (SNR) of the NAA peak was equal to or greater than 15 and the spectral line width was less than 20 Hz. Nine spectra from participants in the FXS group and seven spectra from participants in the comparison group were eliminated, resulting in a final sample size of 18 participants with FXS (14 females) and 18 participants in the comparison group (ten females). These smaller groups did not differ in age, sex or general intelligence (*P*s >0.10), and all subsequent reporting includes only this final sample (Table 1).

**Table 1:** Cognitive measures and medications

**Measure**	**Group**	**N**	**Mean**	**Minimum**	**Maximum**	**SD**
Age	FXS	18	20.54	15.39	25.85	3.57
	Comparison	18	19.80	16.59	25.85	2.61
Verbal IQ	FXS	18	80.22	46	119	21.08
	Comparison	18	72.56	46	100	18.77
Performance IQ	FXS	18	72.78	48	118	18.46
	Comparison	18	76.89	55	121	20.36
Full Scale IQ	FXS	18	74.56	44	119	20.61
	Comparison	18	72.72	48	111	19.68
Contingency Naming Test (CNT)	FXS	16	17.73	6.61	47.65	10.32
	Comparison	16	17.02	4.29	36.00	9.93
Aberrant Behavior Checklist	FXS	15	19.33	0	90	24.85
	Comparison	17	19.65	0	74	19.68
Achenbach attention	FXS	17	59.65	50	83	9.36
	Comparison	17	62.35	50	75	6.70
*Medications*		N	Percent (%)			
SSRI	FXS	4	22.22			
	Comparison	3	16.67			
Stimulants	FXS	2	11.11			
	Comparison	7	38.88			
Other	FXS	4	22.22			
	Comparison	0	0			
Medication-free	FXS	10	27.78			
	Comparison	10	27.78			

Standard scores are reported for Verbal, Performance and Full Scale IQ; higher scores indicate better performance. The CNT score is the number of items correct (out of 27) divided by the time to completion; higher scores indicate better performance. Raw scores are reported for the Aberrant Behavior Checklist; higher scores indicate greater prevalence of aberrant behavior. Achenbach attention refers to the Adult or Child Behavior Checklist attention subscale, T scores are reported for this test (range = 1 to 100, mean = 50); higher scores indicate greater attention problems. *CNT* Contingency Naming Test, *FXS* fragile X syndrome, *IQ* intelligence quotient, Other, atypical antipsychotics, anticonvulsants and other drugs affecting neurological functioning; *SSRI* selective serotonin reuptake inhibitor.

The coordinate location of the MRS voxel was used to prescribe a corresponding 1.5 cm isotropic voxel on each participant’s T2-weighted anatomical image. Brain extraction and segmentation tools from the Oxford Centre for Functional MRI of the Brain (Oxford, UK; FSL, http://fsl.fmrib.ox.ac.uk/fsl/fslwiki/) were used to segment each T2-weighted image, and calculate the percentage of each tissue type (grey matter, white matter, CSF) within each MRS voxel.

Our primary analysis compares metabolite ratios relative to creatine (3.03 ppm), but we also report absolute values as a secondary analysis. A general linear model framework was employed to evaluate group differences in metabolite concentrations and performance on cognitive/behavioral assessments. Our primary goal was to compare group differences in choline and glutamate-related metabolites; comparisons of NAA and *myo*-inositol are included as exploratory analyses. Thus, multiple comparison correction was not warranted. Although the number of participants scanned with each head coil type did not differ between groups (χ^2^ = 0.468, *P* >0.10), head coil type was related to within group metabolite concentration and was therefore added as a covariate in analyses of metabolite concentration.

The MRS voxel contained a greater proportion of grey matter (*P* = 0.015) and a correspondingly smaller proportion of CSF (*P* = 0.035) for individuals with FXS relative to individuals in the comparison group; white matter proportions did not differ (*P* >0.10, Table 2). SNR and line width did not differ between groups (*P*s >0.10). The model assessing group differences in metabolite concentration ratios included group (FXS versus comparison) as the independent variable, metabolite concentration ratio as the dependent variable, and head coil type and grey matter percentage as covariates. Due to the high variability in absolute metabolite values we used the non-parametric Mann–Whitney U test, and thus were unable to account for head coil type and grey matter percentage as covariates at the group level for these absolute values, but metabolite concentrations were normalized for voxel tissue fractions at the individual level. We assessed within group relationships between metabolite concentration ratios and cognitive/behavioral assessment scores with two-tailed Pearson’s correlations.

**Table 2:** Metabolite concentrations

**Metabolite concentrations**	**Group**	**Mean**	**SD**
NAA/creatine	FXS	1.23	0.15
	Comparison	1.38	0.24
Glutamate/creatine	FXS	0.19	0.13
	Comparison	0.20	0.11
Choline/creatine^a^	FXS	0.78	0.11
	Comparison	0.87	0.14
*myo*-inositol/creatine	FXS	0.14	0.05
	Comparison	0.13	0.06
Glx/creatine^a^	FXS	0.20	0.10
	Comparison	0.26	0.10
*Voxel composition (% total volume)*			
Grey matter^a^	FXS	0.35	0.03
	Comparison	0.33	0.02
White matter	FXS	0.34	0.04
	Comparison	0.33	0.02
Cerebrospinal fluid (CSF)^a^	FXS	0.31	0.06
	Comparison	0.34	0.03

^a^Significant group difference. *CSF* cerebrospinal fluid, *FXS* fragile X syndrome, *Glx* glutamine + glutamate, *NAAN*-acetylaspartate.

## Results

All results are presented for the 18 participants in each group with useable spectra. Age, cognitive test scores and parent reports of behavior did not differ between groups (all *P*s >0.10, Table 1). Of this final set of participants, eight individuals in each group were taking medications in one or more of the classes listed: 1) stimulants; 2) SSRIs; and 3) atypical antipsychotics, anticonvulsants and other drugs affecting neurological functioning. The number and percentage of individuals in each group taking each class of medication is presented in Table 1. Within the FXS group, three individuals were taking SSRIs only, five individuals were taking medications in class 3 only, two individuals were taking both stimulants and SSRIs, and three individuals were taking both SSRIs and medications in class 3. Within the comparison group, five individuals were taking stimulants only, one individual was taking an SSRI only, one individual was taking a medication in class 3 only, and three individuals were taking both stimulants and SSRIs.

**Figure 3:**
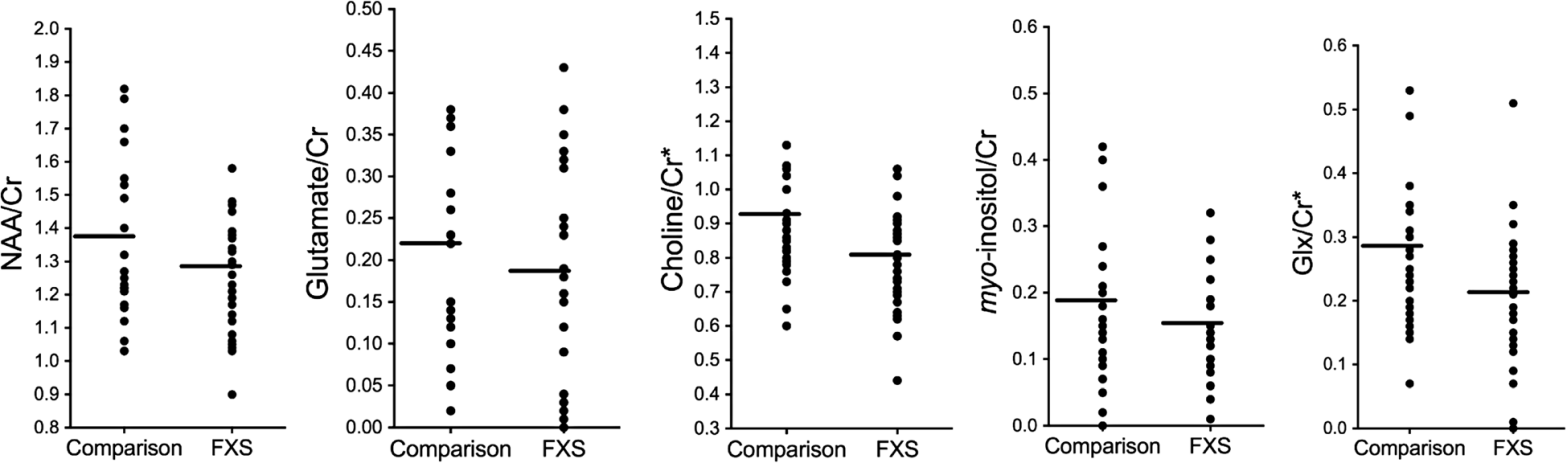
**Metabolite concentration by group.** Metabolite concentrations are in arbitrary units, relative to creatine. * indicates significant group difference. FXS group N = 18; comparison group N = 18. Group means are indicated by horizontal line. FXS, fragile X syndrome; NAA, *N*-acetylaspartate.

We observed reduced choline/creatine ratios (*P* = 0.027) and Glx/creatine ratios (*P* = 0.032) in the FXS group relative to the comparison group (Table 2, Figure 3). There was a trend for reduced NAA/creatine in the FXS group (*P* = 0.082); glutamate/creatine and *myo*-inositol/creatine concentrations did not differ (*P*s >0.10). The pattern of results and significance did not change with the individual taking donepezil excluded: the FXS group displayed reduced choline/creatine (*P* = 0.015) and Glx/creatine (*P* = 0.025); NAA/creatine, glutamate/creatine and *myo*-inositol/creatine levels did not differ (*P*s >0.10). When comparing absolute values we observed reduced choline (*P* = 0.010), Glx (*P* = 0.031) and NAA (*P* = 0.012) in the FXS group relative to the comparison group. Glutamate, *myo*-inositol and creatine concentrations did not differ (*P*s >0.10).

Choline/creatine and Glx/creatine ratios were also compared between female only subgroups (14 females in FXS group, ten females in comparison group), which did not differ in age or general intellectual functioning (*P*s >0.10). For females only, the FXS group displayed significantly lower choline/creatine (*P* = 0.027) and Glx/creatine (*P* = 0.043) levels relative to the comparison group. Statistical analyses were not undertaken for male subgroups due to sample size (four males in FXS group, eight males in comparison group), although effect sizes for between group differences in choline/creatine and Glx/creatine levels were similar to those for females (choline/creatine Glass’s Δ female = 0.569, male = 0.513; Glx/creatine Glass’s Δ female = 0.546, male = 0.604).

Within group analysis of medication effects on each metabolite ratio indicated that metabolite concentration was not significantly related to medication status in either group (all *P*s >0.10). Group comparisons of metabolite concentration were repeated including only medication-free individuals (ten individuals in each group). Choline/creatine and Glx/creatine levels were lower for the FXS group, but the differences did not reach significance (*P*s >0.10).

As an exploratory analysis we examined within group correlations between metabolites for which we found a significant group difference – choline/creatine and Glx/creatine – age, and cognitive/behavioral scores. There were no significant correlations within either group (all *P*s >0.10); results did not change when excluding the participant taking donepezil (*P*s >0.10).

## Discussion

The present study employed single-voxel MRS to examine *in vivo* neurometabolite concentrations in humans with FXS and provides direct evidence of altered metabolite concentration in the caudate nucleus. We demonstrate significantly reduced levels of choline/creatine and Glx/creatine in a group of males and females with FXS, relative to a group of individuals without FXS who were matched for age, sex and general intellectual functioning. These results are in line with the only previously published human FXS MRS study [15] and they corroborate previous reports of altered neurometabolic functioning in animal models of FXS [16]. Aberrant neurometabolite levels may underlie some of the clinical symptoms seen in FXS and they may be related to aberrant receptor signaling seen in animal models [17,18].

FXS has previously been associated with greatly enlarged caudate size [10-12] and aberrant frontostriatal executive functioning networks [14,24]. We provide evidence for altered metabolite concentrations, further elucidating atypical caudate neurobiology in FXS. Given the caudate’s role in learning, memory and executive functions [13], aberrant metabolite levels in this region may mediate some of the behavioral and cognitive deficits associated with FXS. Although the precise effects of FMRP on neurometabolism are not fully understood, recent findings indicate that lack of FMRP results in aberrant functioning of specific GPCRs, mAChRs and mGluRs [17,18], which are highly expressed in striatal circuits [35]. Therefore, the altered neurometabolite levels reported here may be related to hypersensitive mAChR and mGluR signaling. Additionally, FMRP plays a role in regulating calcium-dependent potassium (BK) channels, which are highly expressed in striatal circuits and may also contribute to altered metabolite levels [36]. The direct causal pathway between hypersensitive receptor functioning, BK channel dysregulation and decreased metabolite levels revealed by MRS has yet to be determined, but our results provide an important, although indirect, link.

Glutamate, glutamine and GABA contribute to the Glx peak at 3.78 ppm, although the contribution of GABA is extremely small [37]. Glutamate and glutamine levels are indirectly related to glutamatergic signaling, which is critical for synaptic plasticity and learning [38]; thus, decreased Glx may be a biomarker for learning deficits associated with FXS. A pilot study examining premutation carriers of the *FMR1* gene did not find glutamatergic abnormalities in this condition [39], which is associated with between 55 to 200 CGG repeats and generally normal, though potentially variable overall FMRP production [40]. However, decreased levels of MRS visible Glx have been reported for individuals with autism spectrum disorders (ASD) [41], a set of behaviorally defined disorders in which cognitive and behavioral symptoms overlap with those observed in FXS [42]. As with FXS, animal models of ASD have revealed functional abnormalities in both excitatory (glutamate) and inhibitory (GABA) systems [43-45]. These findings suggest some degree of common neurobiological alteration despite differential origin for cognitive and behavioral symptoms in FXS (reduced FMRP) and idiopathic ASD (variable unknown causes). MRS examinations of ASD have reported decreased levels of NAA [46,47], which has not been previously shown in FXS, although we did report a trend for lower NAA/creatine ratios and lower absolute NAA in FXS. Future studies comparing ASD to FXS directly may be needed to understand common and divergent neurobiological underpinnings.

The MRS visible choline peak at 3.22 ppm includes phosphocholine and glycerophosphocholine, phospholipids involved in membrane synthesis and integrity, which are markers of cellular density [48]. Decreased choline within the FXS group may be indicative of decreased overall cellular density in the caudate. Free choline, the precursor for acetylcholine, represents a relatively small portion of the MRS visible choline peak, yet this peak correlates with *in vivo* acetylcholine measured in rat brain [49]. This animal research suggests reduced choline may indicate altered acetylcholine levels in humans, but more evidence is needed to determine the reliability of MRS signal as a marker of acetylcholine level. Such a non-invasive marker would be extremely useful for the study of FXS given the evidence for altered acetylcholine receptor signaling in *Fmr1*-KO mice [18].

Our primary results suggest that choline and Glx differences are present in both males and females with FXS. Analysis for females only confirms that females with FXS have significantly reduced choline and Glx which, in context with previous research demonstrating altered metabolite levels in males [15], indicates that these neurometabolic systems may be viable candidates for pharmacological treatment endpoints in both sexes. We did not have a large enough sample of male participants to draw conclusions regarding males, but similar effect sizes for male and female participants indicate that similar altered metabolite concentration may exist in both sexes. Future studies with larger sample sizes in each sex are essential for expanding knowledge in this area.

We explored the relationship between neurometabolite concentration and cognitive/behavioral functioning within each group, but found no significant correlations. The measures of cognitive/behavioral functioning we utilized may not have been sensitive enough to detect such relationships and we did not include specific measures of learning or memory, which may be related to choline [50] and glutamine [38] metabolism. Furthermore, sex differences or medication usage may have obscured the relationship between cognitive/behavioral functioning and metabolite concentration. Larger sample sizes, wider age ranges and longitudinal data points are required to clearly elucidate such complex brain/behavior relationships.

The nature of our study population dictated inclusion of participants taking medication and, although there was no within group relationship between metabolite concentration and medication usage, we cannot rule out the possibility that medication has some effect on metabolite concentration. Our *post hoc* analysis including only medication-free individuals showed a trend for lower choline/creatine and Glx/creatine for the FXS group, but differences did not reach significance. Including only medication-free individuals biased our sample toward higher functioning individuals in each group and reduced the statistical power. Larger-scale investigations are required to adequately address the relationships among metabolite concentration, medication usage and phenotypes associated with FXS.

We present metabolite data referenced to creatine, a metabolite widely used as a reference in human MRS, because its concentration remains stable regardless of changes in energy metabolism or disease progression [51], although research suggests creatine levels may be altered in the *Fmr1*-KO mouse [16]. Therefore, we conducted a secondary analysis using absolute water referenced values for each metabolite and noted significant group differences in choline and Glx, as well as in NAA. We interpret the difference in NAA with caution, since we were not able to account for group level covariates in the analysis of absolute concentration and we noted only a trend for lower values in the FXS group on the NAA/creatine ratio. Importantly, we did not find a significant group difference in creatine, supporting the use of that metabolite as a reference in our analysis. We were unable to quantify GABA or glutamine concentrations individually, or to examine more than one region of interest, given our limited time frame for MRI data acquisition. Future investigations employing higher magnet strength, spectral editing and multi-voxel imaging may further elucidate the neurometabolic alterations in FXS.

## Conclusions

We have demonstrated a significant decrease in choline and a combined measure of glutamate and glutamine in the caudate of individuals with FXS, as compared to individuals matched for age, sex and intellectual functioning. These findings corroborate previous reports that FXS is associated with deficits in choline and glutamate-related neurometabolites. Further research is required to determine the exact causal pathway between limited FMRP and altered neurometabolism, as well as the relationship between *in vivo* metabolite concentrations and hypersensitive cholinergic and glutamatergic receptor functioning reported in animal models. Identification of the specific neurometabolic changes involved in FXS dysfunction could produce critical biomarkers for utilization in disease-specific pharmacological treatments. Targeted pharmacological treatments aimed at correcting the neurometabolic system deficits associated with FXS would represent an immense improvement over current therapies used to ameliorate behaviors associated with the disorder. Our results and animal research [16] suggest multiple neurotransmitter system involvement; thus, more than one targeted treatment may be required to adequately address all the behavioral and cognitive issues associated with FXS. Neurobiological imaging modalities such as MRS may help elucidate mechanisms and neural circuits by which absent or reduced FMRP relates to the behavioral and cognitive deficits associated with FXS.

## Abbreviations

ASD: Autism spectrum disorders; CSF: Cerebrospinal fluid; CT-PRESS: Constant-time point-resolved spectroscopy; CNT: Contingency naming test; TE: Echo time; ERK: Extracellular signal-regulated kinase; FOV: Field of view; FMR1: Fragile X mental retardation 1; FMRP: Fragile X mental retardation protein; FXS: Fragile X syndrome; fMRI: Functional magnetic resonance imaging; GPCR: G protein coupled receptors; GABA: Gamma-aminobutyric acid; Glx: Glutamine + glutamate; IQ: Intelligence quotient; KO: Knockout; MRI: Magnetic resonance imaging; MRS: Magnetic resonance spectroscopy; mTOR: Mammalian target of rapamycin; mGluR: Metabotropic glutamate receptor; mGluR1: Metabotropic glutamate receptor 1; mGluR5: Metabotropic glutamate receptor 5; mAChR: Muscarinic acetylcholine receptor; NAA: *N*-acetylaspartate; TR: Repetition time; SSRI: Selective serotonin reuptake inhibitor; SNR: Signal-to-noise ratio.

## Competing interests

ALR disclosed consulting for Novartis (Basel, Switzerland) for biomarkers of FXS based on neuroimaging data. JLB, EWS, EMQ, MR, SP, DS, DM, MG and AAL reported no biomedical financial interests or potential conflicts of interest.

## Authors’ contributions

JLB carried out the statistical analysis and drafted the manuscript. ALR conceived of the study, participated in its design and coordination, and helped to draft the manuscript. EWS, EMQ, MR, SP and AAL recruited participants, collected the data, and helped to analyze the data and draft the manuscript. DS, DM and MG contributed to MRS pulse sequence design, spectroscopy data analysis and interpretation. All authors read and approved the final manuscript.
